# Comparative genomics of *Mycoplasma pneumoniae* isolated from children with pneumonia: South Korea, 2010–2016

**DOI:** 10.1186/s12864-019-6306-9

**Published:** 2019-11-29

**Authors:** Joon Kee Lee, Moon-Woo Seong, Dongjin Shin, Jong-Il Kim, Mi Seon Han, Youbin Yeon, Sung Im Cho, Sung Sup Park, Eun Hwa Choi

**Affiliations:** 1 Department of Pediatrics, Seoul National University College of Medicine, Seoul, South Korea; 2 Department of Pediatrics, Chungbuk National University Hospital, Cheongju, South Korea; 3 Department of Laboratory Medicine, Seoul National University Hospital, Seoul National University College of Medicine, Seoul, South Korea; 4 Biomedical Research Institute, Seoul National University Hospital, Seoul, South Korea; 5 Department of Biomedical Sciences, Seoul National University Graduate School, Seoul, South Korea; 6 Department of Biochemistry and Molecular Biology, Seoul National University College of Medicine, Seoul, South Korea; 7 Genomic Medicine Institute, Medical Research Center, Seoul National University, Seoul, South Korea; 8 Department of Pediatrics, Seoul National University Children’s Hospital, 101 Daehak-ro, Jongno-gu, Seoul, 03080 South Korea

**Keywords:** *Mycoplasma pneumoniae*, Whole genome analysis, Comparative genomics

## Abstract

**Background:**

*Mycoplasma pneumoniae* is a common cause of respiratory tract infections in children and adults. This study applied high-throughput whole genome sequencing (WGS) technologies to analyze the genomes of 30 *M. pneumoniae* strains isolated from children with pneumonia in South Korea during the two epidemics from 2010 to 2016 in comparison with a global collection of 48 *M. pneumoniae* strains which includes seven countries ranging from 1944 to 2017.

**Results:**

The 30 Korean strains had approximately 40% GC content and ranged from 815,686 to 818,669 base pairs, coding for a total of 809 to 828 genes. Overall, BRIG revealed 99% to > 99% similarity among strains. The genomic similarity dropped to approximately 95% in the P1 type 2 strains when aligned to the reference M129 genome, which corresponded to the region of the *p1* gene. MAUVE detected four subtype-specific insertions (three in P1 type 1 and one in P1 type 2), of which were all hypothetical proteins except one tRNA insertion in all P1 type 1 strains. The phylogenetic associations of 30 strains were generally consistent with the multilocus sequence typing results. The phylogenetic tree constructed with 78 genomes including 30 genomes from Korea formed two clusters and further divided into two sub-clusters. eBURST analysis revealed two clonal complexes according to P1 typing results showing higher diversity among P1 type 2 strains.

**Conclusions:**

The comparative whole genome approach was able to define high genetic identity, unique structural diversity, and phylogenetic associations among the 78 *M. pneumoniae* strains isolated worldwide.

## Background

*M. pneumoniae* is an important cause of respiratory tract infections in children and adults, ranging from mild upper respiratory infections to life-threatening conditions [1]. *M. pneumoniae* infections are more common among children 5 years of age or older than among younger children [2]. Mild upper respiratory infections are common with a considerable portion of asymptomatic patients, but 3 to 10% develop pneumonia with a wide spectrum of radiologic findings [3–5]. Extrapulmonary abnormalities are an important part of *M. pneumoniae* diseases both in diagnosis and treatment. The spectrum of manifestations includes extrapulmonary symptoms such as skin rash, hemolytic anemia, arthritis, and neurologic abnormalities [1].

P1 adhesin (P1), a 170-kD surface protein located at the tip-like structure of virulent *M. pneumoniae*, mediates its cytoadherence to the surface of respiratory epithelial cells [6]. As P1 adhesin protein plays a critical step in the infection process, studies regarding the genetics of *M. pneumoniae* focused mainly on P1 types and subtypes [7, 8]. P1 typing was the only available tool that could be applied in the past to determine genotype. Although P1 typing can separate *M. pneumoniae* into two types and additional six variants, it did not always convey information regarding epidemiologic characteristics or clinical severity. New genetic analysis techniques, such as multilocus variable-number tandem-repeat analysis (MLVA) and multilocus sequence typing (MLST), have been applied to *M. pneumoniae* [9, 10].

Despite the evolution of molecular microbiology and advanced classifications beyond P1 typing, research to understand the entire genome structures of *M. pneumoniae* in regard to molecular epidemiology has remained much behind that of other bacteria such as *Streptococcus pneumoniae* and *Escherichia coli*. Recent advances in molecular microbiology and bioinformatics have made it possible to analyze *M. pneumoniae* through high-throughput sequencing technologies such as Illumina dye sequencing, pyrosequencing, and single-molecule real-time (SMRT) sequencing [11]. The whole genome of *M. pneumoniae* is ≈820 kb and has up to 700 coding operons [12]. The comparably small genome size and limited number of operons are challenges in the genomic investigation of *M. pneumoniae*.

This study aims to analyze genomes of 30 *M. pneumoniae* strains isolated from children with pneumonia in South Korea during two epidemics from 2010 to 2016 and compare with a global collection of 48 *M. pneumoniae* strains which includes seven countries ranging from 1944 to 2017.

## Results

### Strain characteristics

The strains were isolated from nasopharyngeal samples obtained from children with pneumonia. Thirty-seven and 45 *M. pneumoniae* strains were collected in 2010–12 and 2014–16, respectively. Thirty *M. pneumoniae* strains were chosen for the current study (Additional file 1). Eighteen strains and twelve strains were selected from 2010 to 12 and 2014–16 epidemic years, respectively. Twenty-four (80.0%) P1 type 1 strains, five (16.7%) P1 type 2c strains and a P1 type 2a strain (3.3%) were included. Five sequence types (STs) were included: ST1 (*n* = 2, 6.7%), ST3 (*n* = 20, 66.7%), ST14 (*n* = 5, 16.7%), ST17 (*n* = 2, 6.7%), and ST33 (*n* = 1, 3.3%).

### Genome assembly

The characteristics of the assemblies and the background information are found in Table 1. The resulting contigs were mapped to the M129 reference genome and joined via PCR. The thirty genomes had all contigs joined to form a single, continuous (circular) contig. Following assembly and editing, the genomes underwent automated gene annotation. With approximately 40% GC content and ranging from 815,686 to 818,669 bp, the genomes coded for a total of 809 to 828 genes.

**Table 1:** Genome lengths and contigs determined from the initial assembly with complete genome structures annotated by RAST

Strain	Contigs	L50	N50	Min Length	Max Length	Total Length	%GC	Genes		
								CDS	RNA	Total
10–980	6	2	152,732	14,538	390,907	816,424	40.0	776	40	816
10–1048	6	2	152,735	14,538	392,185	816,465	40.0	777	40	817
10–1059	7	2	98,837	14,538	392,164	816,681	40.0	776	40	816
10–1110	8	2	152,733	20,993	388,970	816,522	40.0	775	40	815
10–1213	5	1	451,397	14,538	451,397	816,521	40.0	772	40	812
10–1257	3	1	702,439	14,562	702,439	816,333	40.0	776	40	816
10–1385	9	3	95,255	14,577	297,117	817,191	40.0	780	39	819
11–107	5	2	249,794	14,538	389,683	816,346	40.0	773	40	813
11–129	6	2	152,693	14,538	392,172	816,432	40.0	775	40	815
11–174	6	2	258,682	13,367	282,196	815,686	40.0	776	39	815
11–212	7	2	152,734	14,538	389,655	816,503	40.0	778	40	818
11–473	6	2	152,734	14,538	389,647	816,518	40.0	778	40	818
11–634	7	2	152,735	14,775	391,525	816,551	40.0	777	40	817
11–949	6	2	258,658	13,367	283,608	817,102	40.0	784	39	823
11–994	5	2	249,776	14,538	389,685	816,304	40.0	776	40	816
11–1384	6	2	258,694	13,367	283,575	818,669	40.0	787	39	826
12–060	6	2	152,734	14,538	392,205	816,506	40.0	775	40	815
12–091	6	2	152,734	14,538	391,968	816,510	40.0	777	40	817
14–637	6	2	156,124	60,136	298,090	818,560	40.0	789	39	828
15–215	6	2	152,734	14,561	392,183	816,388	40.0	775	40	815
15–885	6	2	152,734	14,561	389,671	816,420	40.0	776	40	816
15–969	6	2	152,735	14,538	392,144	816,389	40.0	780	40	820
15–982	5	2	156,554	14,538	390,947	816,495	40.0	769	40	809
16–002	6	2	152,736	14,538	389,658	816,530	40.0	773	40	813
16–004	6	2	152,736	14,538	392,133	816,561	40.0	777	40	817
16–032	6	2	152,734	14,538	392,119	816,471	40.0	772	40	812
16–118	5	1	443,549	14,538	443,549	816,467	40.0	775	40	815
16–462	5	2	152,735	57,889	392,162	816,525	40.0	776	40	816
16–710	7	2	152,734	14,538	392,162	816,537	40.0	773	40	813
16–734	6	2	258,694	13,367	283,522	818,445	40.0	784	39	823

L50, smallest number of contigs whose length sum makes up half of genome size; N50, sequence length of the shortest contig at 50% of the total genome length; CDS, coding sequence

### Overall comparison

**Fig. 1:**
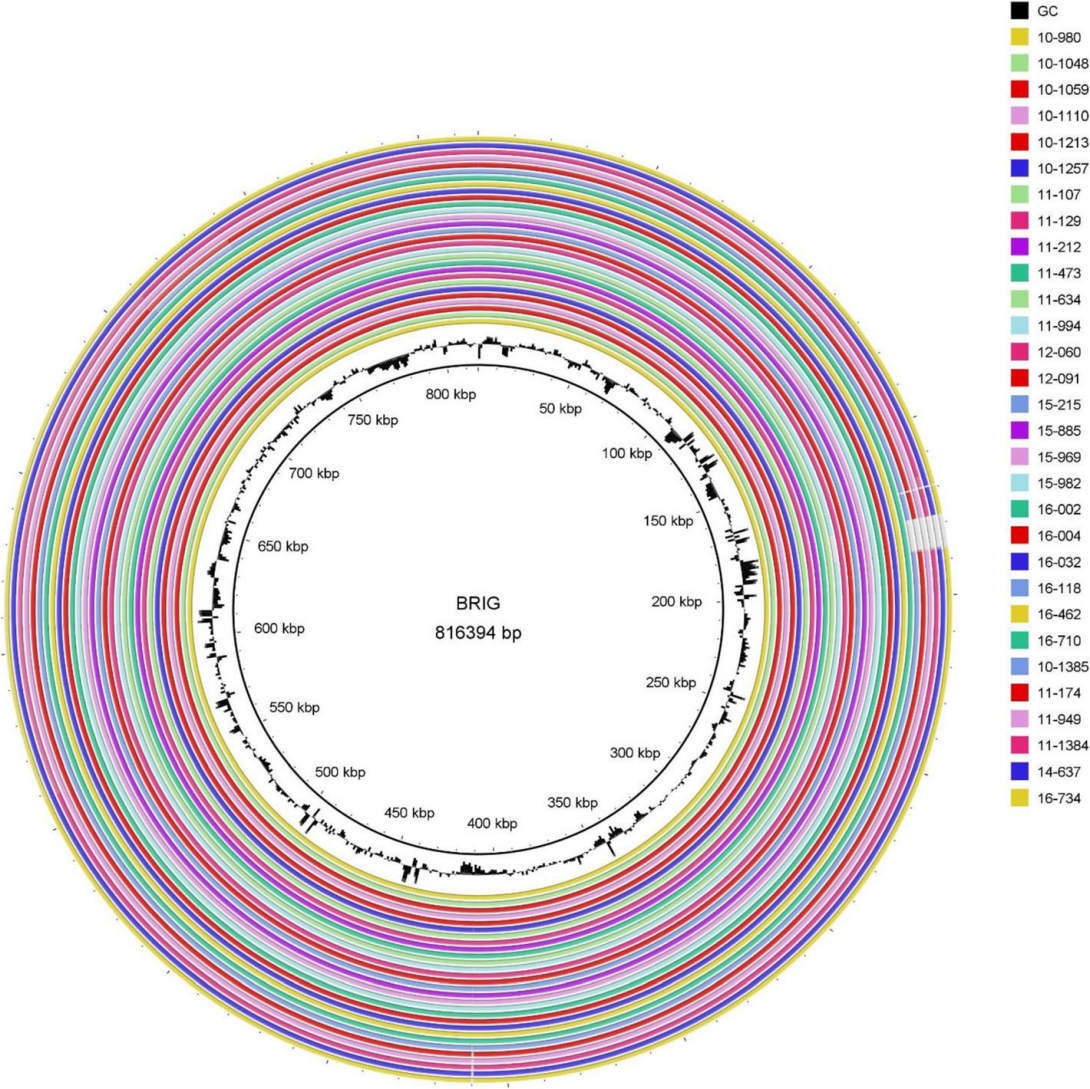
Overall sequence identity of the 30 sequenced strains with the reference M129 genome. Solid coloration indicates > 99% identity and transparent grey indicates approximately 95% identity. Location in the reference genome is indicated by numeration on the inside of the ring. GC content in the reference genome is indicated by the black bar graphs between the genomic coordinates and the colored rings (bars pointing toward the outside of the circle indicate high GC content)

The 30 sequenced genomes were aligned to the reference M129 genome using BLAST Ring Image Generator (BRIG). Overall, the genomes were 99% to > 99% identical. The similarity dropped to approximately 95% in the type 2 strains, which corresponded to the area of the *p1* gene (Fig. 1).

### Genomic structural comparison

**Fig. 2:**
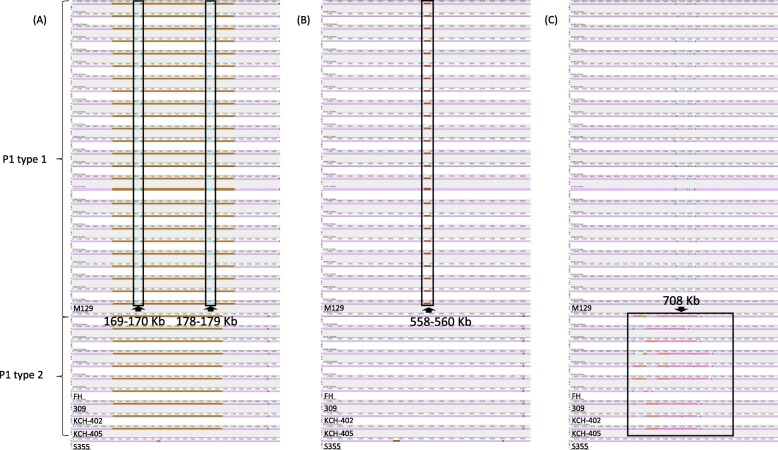
Whole genome alignment of the 30 sequenced strains with 6 reference sequences using MAUVE. Regions colored in MAUVE are conserved across all strains. **a** Two 1 Kbp (approximate) insertions are noticed in the P1 type 1 groups at 169–170 Kb and 178–179 Kb. **b** A 2 Kbp (approximate) insertion is noticed in the P1 type 1 groups at 558–560 Kb. **c** A 6 Kbp (approximate) insertion is noticed in the P1 type 2 groups at 708 Kb. All positions are based on M129 reference strain

For the detection of large chromosomal rearrangements, deletions, and duplications, MAUVE was applied to the 30 sequenced genomes with 6 reference genomes. All genomes fell into three locally collinear blocks (LCBs), which are conserved segments. The three LCBs were in the same order without any rearrangement. MAUVE detected four subtype-specific insertions (Fig. 2): three type 1-specific insertions (M129 numbering; 169–170 kb, 178–179 kb, and 558–560 kb) and a type 2-specific insertion (M129 numbering; 708 kb). The subtype-specific insertions were manually annotated. Type 1 insertions were all annotated as hypothetical proteins (MPN130, MPN137, MPN138, and MPN457–459) except for the tRNA gene (MPNt26) positioned at 558635 to 558,723 (M129 numbering). The proteins of the type 2 insertion (6 kbp) were annotated as hypothetical proteins without exception (BIX66_03340, 03345, 03350, 03355, and 03360).

### SNP and indel analysis

SNPs and indels were compared for the identification of sequence level differences against the reference genome. The results are shown in Table 2. As expected, P1 type 1 strains showed fewer variant numbers (140–455) than P1 type 2 strains (1778–1796), showing a clear distinction.

**Table 2:** Variant patterns relative to the nucleotide and amino acid structure of M129 reference strain

	Upstream	Synonymous	Missense	Splice	Start/stop	In-frame	Frameshift	Total
10–980	37	32	48		4	3	16	140
10–1048	89	105	153		13	6	25	391
10–1059	93	100	149		11	7	29	389
10–1110	56	31	49		5	2	16	159
10–1213	93	102	154		16	7	25	397
10–1257	92	95	151		15	5	25	383
10–1385	518	480	659	1	56	9	55	1778
11–107	114	107	172		15	9	23	440
11–129	96	113	160		13	6	28	416
11–174	518	479	658	1	57	11	54	1778
11–212	118	108	154		13	7	25	425
11–473	116	97	141		15	5	25	399
11–634	110	103	154		16	6	25	414
11–949	521	489	665	1	53	9	55	1793
11–994	92	99	151		12	7	24	385
11–1384	519	490	668	1	53	9	56	1796
12–060	119	104	160		15	7	25	430
12–091	130	104	162		16	7	27	446
14–637	518	483	657	1	51	11	59	1782
15–215	95	106	155		13	7	27	403
15–885	130	108	170		15	7	25	455
15–969	114	104	157		14	8	25	422
15–982	142	108	157		14	8	25	454
16–002	92	104	156		12	8	25	397
16–004	116	114	163		14	8	27	442
16–032	121	106	166		17	6	25	441
16–118	126	100	156		14	7	25	428
16–462	128	101	159		14	7	25	434
16–710	115	100	158		14	7	25	419
16–734	519	486	660	1	54	10	55	1785

### Proteins and functional analysis

**Fig. 3:**
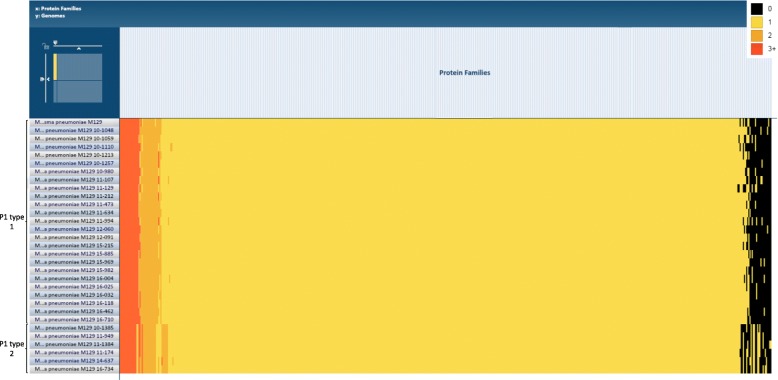
Heatmap of protein families of 30 sequenced genomes with reference genome *M. pneumoniae* M129. Cell color represents the number of proteins from a specific genome in a given protein family. Note that P1 types 2 (10–1385, 11–174, 11–949, 11–1384, 14–637 and 16–734) are distinguishable from P1 types 1

The Protein Family Sorter tool at Pathosystems Resource Integration Center (PATRIC) allows selection of a set of genomes of interest and examination of the distribution of protein families across genomes. An interactive heatmap viewer provides a comprehensive view of the distribution of the protein families across multiple genomes, with clustering and anchoring functions to show relative conservation of synteny and to identify lateral transfers. Based on gene annotation from PATRIC, a heatmap of all proteins was produced along with the reference genome *M. pneumoniae* M129 (Fig. 3). Unsurprisingly, when genomes were classified into P1 types 1 and 2, distinction between the genomes was apparent. Nevertheless, most of the genomes that showed different expressions were hypothetical proteins with uncertain significance.

### Phylogenetic associations

Thirty genomes were aligned with MAFFT, and a phylogenetic tree was generated (Additional file 2). The phylogenetic tree was divided into two clades in accordance with the P1 typing. In general, the STs of the 30 strains were consistent with the phylogenetic relationship.

**Fig. 4:**
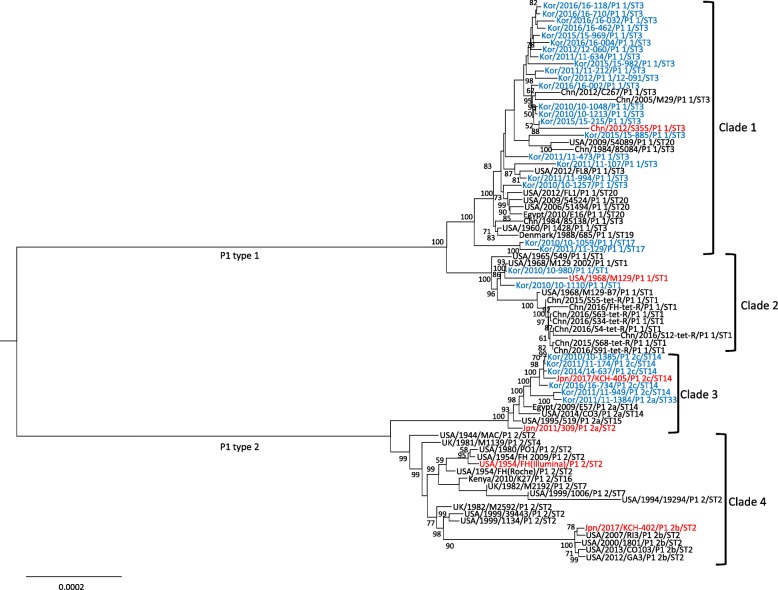
Phylogenetic tree based on whole genome alignment of the 30 sequenced strains with 48 *M. pneumoniae* genomes accessed from NCBI. The tree was built through 500 bootstraps using the maximum composite likelihood approach based on neighbor-joining algorithms. Branch length designates actual distance. Bootstrapping values over 50 are represented on the tree. Blue colored strains are from this study and red colored strains are the 6 references. Strains are grouped into four distinct clades. ST, sequence type

All 78 strains, including strains from this study and NCBI, were aligned and phylogenetic tree was constructed and visualized (Fig. 4). In general, the strains in this study were scattered throughout the entire phylogenic tree, along with the expansion of certain clades. Trees were divided into two major clades in accordance with the P1 typing. Each P1 type was divided into another two clades. Clade 1 formed the largest clade. It included strains of ST3 from the current study and global collections. Strains with ST20, ST17 and ST19 were included in Clade 1. Clade 2 was consisted of ST1 strains, exclusively. This clade harbored a subclade which consisted of strains from China in year 2015 and 2016. Clade 2 also included the M129 reference strain. Major ST of Clade 3 was ST14 with one each of ST2, ST15 and ST33 strain. Clade 4 showed high proportion of ST2 strains with a subclade which included four ST2 strains from USA and a ST2 strain from Japan. Overall, Clade 1 showed the most heterogenicity in terms of both the origin and the time of strain collected.

### Comparative genomics with global strains-MLST

For the comparative genome analysis of global strains, 48 genomes of *M. pneumoniae* were accessed from NCBI. Typing of P1 types and MLST types was performed (Additional file 1). An eBURST diagram was constructed based on the 30 strains from this study, 48 global strains from NCBI, and previously reported STs from PubMLST (http://pubmlst.org/mpneumoniae/).

**Fig. 5:**
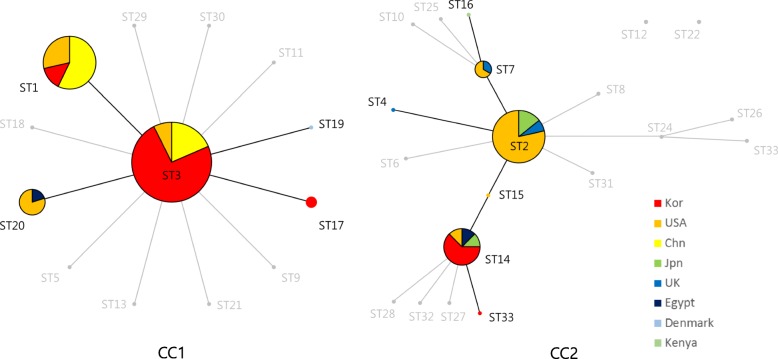
*Mycoplasma pneumoniae* sequence type (ST) relationship by eBURST analysis including 30 strains from this study, 48 strains from NCBI, and previously reported STs from PubMLST (http://pubmlst.org/mpneumoniae/). Two main CCs were defined with two singletons (ST12 and ST22). ST3 and ST2 were the predicted founder of each CC. The size of each circle correlates with the number of isolates of each ST. STs in gray are previously reported, but not included in the investigation of this study. CC, clonal complex

The eBURST diagram showed two clonal complexes with two singletons of ST12 and ST22 (Fig. 5). The founder ST of CC1 was identified as ST3 with no double locus variants (DLVs). The founder ST of CC2 was recognized as ST2 with multiple subgroup founders (ST7, ST14 and ST24), multiple single locus variants (SLVs) and DLVs. In the eBURST diagram of global strains, ST3 and ST1, and ST2 and ST14 were the main STs from CC1 and CC2, respectively. Strains from this study (colored in red) constituted a considerable proportion of ST3 from CC1 and ST14 from CC2. There were several other STs that were previously reported, but not included in the investigation of this study.

## Discussion

*M. pneumoniae* is known as an organism ‘difficult-to-culture’ [1]. Thus, unlike ordinary bacterial pathogens, the aid of molecular biology in the diagnosis of *M. pneumoniae* is critical [13]. As the burden of disease caused by this organism is considerable and patients may experience diverse extrapulmonary clinical manifestations, *M. pneumoniae* has drawn the attention of many researchers. Nevertheless, in addition to the molecular diagnosis of *M. pneumoniae* by the P1 adhesin, P1 typing has been the sole method for classification for decades [14]. However, because the size of the *M. pneumoniae* genome is short compared to that of other bacteria and because the P1 adhesin is the only diverse part of the whole genome, researchers continued to focus on the P1 adhesin. Despite these efforts, P1 was not sufficient for the explanation of epidemics or for the explanation of clinical severity [15, 16].

Recent advances in molecular microbiology have widened the scope of the implementation of sophisticated techniques, such as MLVA and MLST [9, 10]. New classifications developed by such new technologies have expanded P1 classification with enhanced distinction. Nevertheless, epidemics still cannot be clearly explained by the new technologies, and there are reports that chest X-rays are the most predictive clue in the course of infection regardless of the molecular genetics [4]. Nevertheless, attempts to utilize molecular biology by using MLVA or MLST have shown useful insights in understanding epidemiology of *M. pneumoniae*. A recent study from Korea demonstrated high proportion of ST3 in a 16-year period [17]. ST3 was also frequently identified in Japan during the similar period, but ST19 was prevalent among macrolide-resistant strains in Japan, while ST19 has never been identified in Korea [18]. A study from China, which applied MLVA on 835 samples from different regions, has also found regional differences in genotype distribution [19].

Although not extensively applied, high-throughput technologies have been applied to the investigation of *M. pneumoniae*. A study conducted by Xiao et al. analyzed 15 *M. pneumoniae* genomes obtained by Illumina sequencing, including 11 clinical isolates and 4 reference strains (20). Although approximately 1500 SNP and indel variants exist between type 1 and type 2 strains, an overall high degree of sequence similarity was found among the strains (> 99% identical to each other). The study concluded that the *M. pneumoniae* genome is extraordinarily stable over time and geographic distances across the globe, with a striking lack of evidence of horizontal gene transfer.

One of the most recent NGS studies performed by Diaz et al. demonstrated WGS analysis of 107 *M. pneumoniae* isolates, including 67 newly sequenced isolates, using the Pacific BioSciences RS II and/or Illumina MiSeq sequencing platforms [21]. Population structure analysis done by this study supported the existence of six distinct subgroups. Although this study included the largest collection of *M. pneumoniae* isolates ever, only a few strains were included from Asian regions where the unique epidemiologic features (for example, high rate of macrolide-resistance among *M. pneumoniae*) are noticed.

Comparative genome analysis was performed using BRIG, MAUVE, and MAFFT. The genomes were classified mainly by the legendary P1. BRIG clearly distinguished P1 types 1 and 2, but no further information could be found, as separate genes could not be visualized [22]. MAUVE utilizes LCBs, which are conserved segments that appear to be internally free from genome rearrangements [23]. The result from MAUVE showed that large rearrangements (e.g., plasmids, phage or resistance genes) were not observed among *M. pneumoniae*. Specific insertions were noted in both P1 types. Nevertheless, the translated proteins of the inserted genes were generally hypothetical proteins with the exception of a tRNA. This is consistent with a previous report by Xiao et al.*,* but the two insertions at 169–170 kb and 178–179 kb have not been described previously [20]. The heatmap generated by PATRIC confirmed the P1 classification by differences in protein production. This is consistent with additional studies that applied NGS technology [24, 25].

The SNP approach is widely used in the study of antimicrobial resistance and genetic diversity and is not limited to *M. pneumoniae* [26–28]. This study is consistent with previous studies investigating SNPs within *M. pneumoniae* [20, 21]. Variant calling against M129 of P1 subtypes showed substantially fewer variants compared to P1 type 2 in both nonsynonymous SNPs and total variants.

The two phylogenetic trees constructed and visualized in this study revealed notable findings. First, the phylogenetic relatedness of the 30 strains demonstrated strong correlation according to the P1 type. Each ST type was generally grouped by the same branch. Nevertheless, when global strains were considered together, there were a few exceptions which suggests the associations demonstrated by the phylogenetic tree do not fully correlate with the ST type. Examples include ‘Kor/2011/11–1384/P1_2a/ST33’ strain which is placed along with ST14 strains or ‘Kenya/2010/K27/P1_2/ST16’ strain which is placed along with ST2 strains. Second, when eBURST analysis and the phylogenetic associations with global strains are considered together, the correlation of two methods for comparative genomics were apparent. Clade 2 from the phylogenetic tree stands for ST1 strains of CC1 in the MLST analysis. It is highly probable that Clade 1 takes the rest of the strains in CC1. Contiguous strains of the CC2 which includes ST2, ST15, ST14 and ST33 are consistent with strains from Clade 3. In another direction, contiguous strains of the CC2 which includes ST2, ST4, ST7 and ST16 is consistent with strains from Clade 4. Even though not apparent in the phylogenetic analysis probably due to the genetical proximity of the strains, eBURST analysis shows ST3 as the founder strain of the CC1. We assume that despite the fact that M129, one of the ST1 strains, is used as reference strain, it is more convincing that ST1 strains may have evolved from the ST3 strains. The strain ‘USA/1960/P1_1428/P1_1/ST3’ which is the earliest known strain of the P1 type 1 strains also supports this idea.

In general, the result of the current study is consistent with that of the previous studies [20, 21]. High stability was observed by the small number of SNPs across the genome and lack of rearrangements. The fact that P1 types shown as a major factor for the genetic classification is also consistent with the findings of the current study. Diaz et al. grouped 107 strains from four other studies and their study into three P1 type 1 and two P1 type 2 subgroups based on core protein sequences [21]. Even though there are differences in the methods of tree alignment, construction, and visualization, the subgroups are consistent with the current study, in general. A distinct subgroup designated as 1 N (New) which included four isolates from their study was the only subgroup which did not exist on the current study. When comparisons are made between the different phylogenetic trees, we find that the abundance and heterogenicity of the Clade 1 in the current study and the group 1 U (Ubiquitous) in the study of Diaz et al. as the common finding. We assume that this certain subgroup harbors the most actively evolving strains in global and demands attention in terms of pathogenicity or in accordance with macrolide resistance.

This study has some limitations. First, the number of strains included in the study was small, thus we were not able to interpret the clinical significance of the findings. Second, isolates were chosen from two consecutive outbreaks. Further analysis from sporadic cases and new outbreaks is needed. Nevertheless, this study expanded our understanding of the genome structure of *M. pneumoniae* through whole genome analysis. Whole genome approach provided more detailed information than traditional molecular typing methods for exploring genomic diversity among *M. pneumoniae* strains.

## Conclusions

The comparative whole genome approach was able to define high genetic identity, unique structural diversity, and phylogenetic associations among the 78 *M. pneumoniae* strains isolated worldwide.

## Methods

### *M. pneumoniae* strains

This study comprised *M. pneumoniae* strains detected from children with pneumonia at two hospitals during two consecutive outbreaks of *M. pneumoniae* pneumonia in South Korea in 2010–2012 and 2014–2016. Specimens were obtained from Seoul National University Children’s Hospital (Seoul) and Seoul National University Bundang Hospital (Seongnam). Epidemic periods and the diagnosis of *M. pneumoniae* pneumonia were defined as previously described [17].

### Cultivation

Culture of *M. pneumoniae* was performed using pleuropneumonia-like organism broth as previously described [4]. Reference strain M129 (ATCC 29342) was used as a positive control for culture.

### DNA preparation

DNA was extracted directly from cultivated *M. pneumoniae* using an extraction kit (DNeasy Kit; QIAGEN, Hilden, Germany) according to the manufacturer’s instructions. The *p1* gene was amplified by PCR for the confirmation of *M. pneumoniae*.

### MLST analysis and P1 typing

MLST was performed on the *M. pneumoniae* DNA samples as previously described [10]. P1 subtypes and each subtype variants were determined by sequencing the *RepMP2/3* and *RepMP4* genes and in comparison with previously published data [29, 30].

### Selection of strains for whole-genome analysis

A total of 30 strains were selected for the whole-genome sequencing (WGS) investigation. Thirty-seven *M. pneumoniae* strains were isolated during the 2010–2012 epidemic. P1 subtype 1 accounted for 71.9% and ST3 was responsible for 62.2%. The remaining 37.8% consisted of ST1, ST14, ST17, and ST33. In contrast, among the 45 isolates detected during the 2014–2016 epidemic, P1 subtype 1 accounted for 50.0% and the ST distribution was 88.9% for ST3 and 11.1% for ST14. In order to include as many different STs as possible, all strains that showed STs other than ST3 (ST1, ST14, ST17, and ST33) were included for WGS analysis. We have randomly selected 20 ST3 strains from each epidemic.

### Next-generation sequencing (NGS)

The library for whole genome sequencing was prepared using Truseq Nano DNA Lib Prep Kit (Illumina, San Diego, CA, USA) and sequenced using MiSeq Reagent Kit V2 (Illumina, San Diego, CA, USA) on the Illumina MiSeq desktop sequencer (Illumina, San Diego, CA, USA). Illumina NGS workflows include four basic steps: library preparation, cluster amplification, sequencing and alignment. The NGS library is prepared by fragmenting a genomic DNA sample and ligating specialized adapters to both fragment ends. The library is loaded into a flow cell, and the fragments are hybridized to the flow cell surface. Each bound fragment is clonally amplified through bridge amplification. Sequencing repeats, including fluorescently labeled nucleotides, are added, and the first base is incorporated. The flow cell is imaged, and the emission from each cluster is recorded. The emission wavelength and intensity are used to identify the base. This cycle is repeated ‘n’ times to create a read length of ‘n’ bases. In this study, paired-end 250-bp reads were used with an average depth (coverage) of 442.93 (ranging from 172.95 to 795.39). The average number of reads during the sequencing was 1,445,719 (ranging from 564,516 to 2,596,168). Instead of directly aligning the reads to a reference sequence, de novo assembly was performed.

### Genome assembly and annotation

NGS reads were assembled de novo using SPAdes [31]. The number of contigs generated ranged from 3 to 8 per strain. These contigs were mapped to the M129 reference genome using the BLAST-like alignment tool (BLAT) and visualized using Integrative Genomics Viewer (IGV) [32–34]. This mapping was used to develop PCR primers to join the contigs. High fidelity PCRs and Sanger sequencing were performed using standard methods. Overlapping and joining of the contigs were performed manually with Sequencher version 5.4.6 (Gene Codes Corporation, Ann Arbor, MI, USA). The initial NGS reads were aligned to the de novo assembled genome for the correction of errors. The corrected and completed circular genomes were annotated using Rapid Annotation using Subsystem Technology (RAST) [35].

### Comparative genomics

Completed genomes were aligned using BRIG for the overall sequence similarity between the strains [22]. MAUVE was used to detect large chromosomal rearrangements, deletions, and duplications [23]. In the phylogenetic analysis with the 48 global strains downloaded from the National Center for Biotechnology Information (NCBI) were included. MAFFT was applied using the ‘FFT-NS-2’ method for multiple sequence alignment of the strains from the current study and with the global strains. Phylogenetic tree was constructed using the maximum composite likelihood approach based on neighbor-joining algorithms and visualized using Phylo.io (strains from the current study) and MEGA X (with the global strains) [36, 37]. For the phylogenetic tree with the global strains, 500 iterations of bootstrapping analysis were used to generate confidence values. eBURST version 3 software (http://eburst.mlst.net/) was used to estimate the relationships among the strains and to assign strains to a clonal complex (CC) [38].

### Single nucleotide polymorphism (SNP) and insertion/deletion (indel) analysis

To call SNPs and indels, completed genomes were first broken into 10-kb “reads” at 1-kb intervals and then aligned to the M129 reference strain (NCBI Accession Number NC_000912) using BWA v0.7.7 [39]. Variant calling was performed using Samtools [40]. The effects of the SNPs and indels in the resulting VCF files were evaluated and annotated using SnpEff v3.3 [41].

### Proteins and functional analysis

For the analysis of proteins and functional annotation, PATRIC was used, and a heatmap was generated based on annotations [42]. Gene translation, multiple sequence alignment and visualization of proteins were performed using Clustal Omega [43]. Annotation of any hypothetical genes was performed using a BLAST search against the Kyoto Encyclopedia of Genes and Genomes (KEGG) database [44, 45].

### References genomes

Six reference genomes were included in each analysis as appropriate (Table 3). *M. pneumoniae* M129, FH, 309, KCH-402 and K405 are representatives of each P1 type and subtype. *M. pneumoniae* S355 is included, as this strain is one of the earliest strains that was fully sequenced and expressed macrolide resistance. Two FH strains were downloaded from NCBI, and the genome sequenced with Illumina was used as the reference genome.

**Table 3:** Reference genomes included in the analysis

NCBI Accession	Organism	Length (bp)	P1 type	Year Collected	Origin	Description
NC_000912.1	*M. pneumoniae* M129	816,394	1	1968	USA/NC	ATCC 29342 (Reference)
CP_010546.1	*M. pneumoniae* FH	817,207	2	1954	USA/MA	ATCC 15531 (Reference)
NC_016807.1	*M. pneumoniae* 309	817,176	2a	2011	Japan	
AP_017318.1	*M. pneumoniae* KCH-402	817,074	2b	2017	Japan	
AP_017319.1	*M. pneumoniae* KCH-405	817,099	2c	2017	Japan	
CP_013829.1	*M. pneumoniae* S355	801,203	1	2016	China	Macrolide resistant

## Supplementary information

**Additional file 1:** P1 type and MLST type of the 30 strains from this study and 48 strains from NCBI.

**Additional file 2:** Phylogenetic tree based on whole genome alignment of the 30 sequenced strains.

## Data Availability

All data generated or analyzed during this study are included in this published article. The gene sequences are deposited in NCBI database under the accession numbers CP039761-CP039790.

## Abbreviations

BRIG: BLAST Ring Image Generator
CC: clonal complex
DLV: double locus variant
MLST: multilocus sequence typing
MLVA: multilocus variable-number tandem-repeat analysis
PATRIC: Pathosystems Resource Integration Center
SLV: single locus variant
ST: sequence type
