# Supplementary figures and images for: Comparative genomics of Mycoplasma pneumoniae isolated from children with pneumonia: South Korea, 2010–2016
Source: BMC Genomics. 2019 Nov 29;20:910. doi: 10.1186/s12864-019-6306-9 (PMC6884898; doi:10.1186/s12864-019-6306-9)

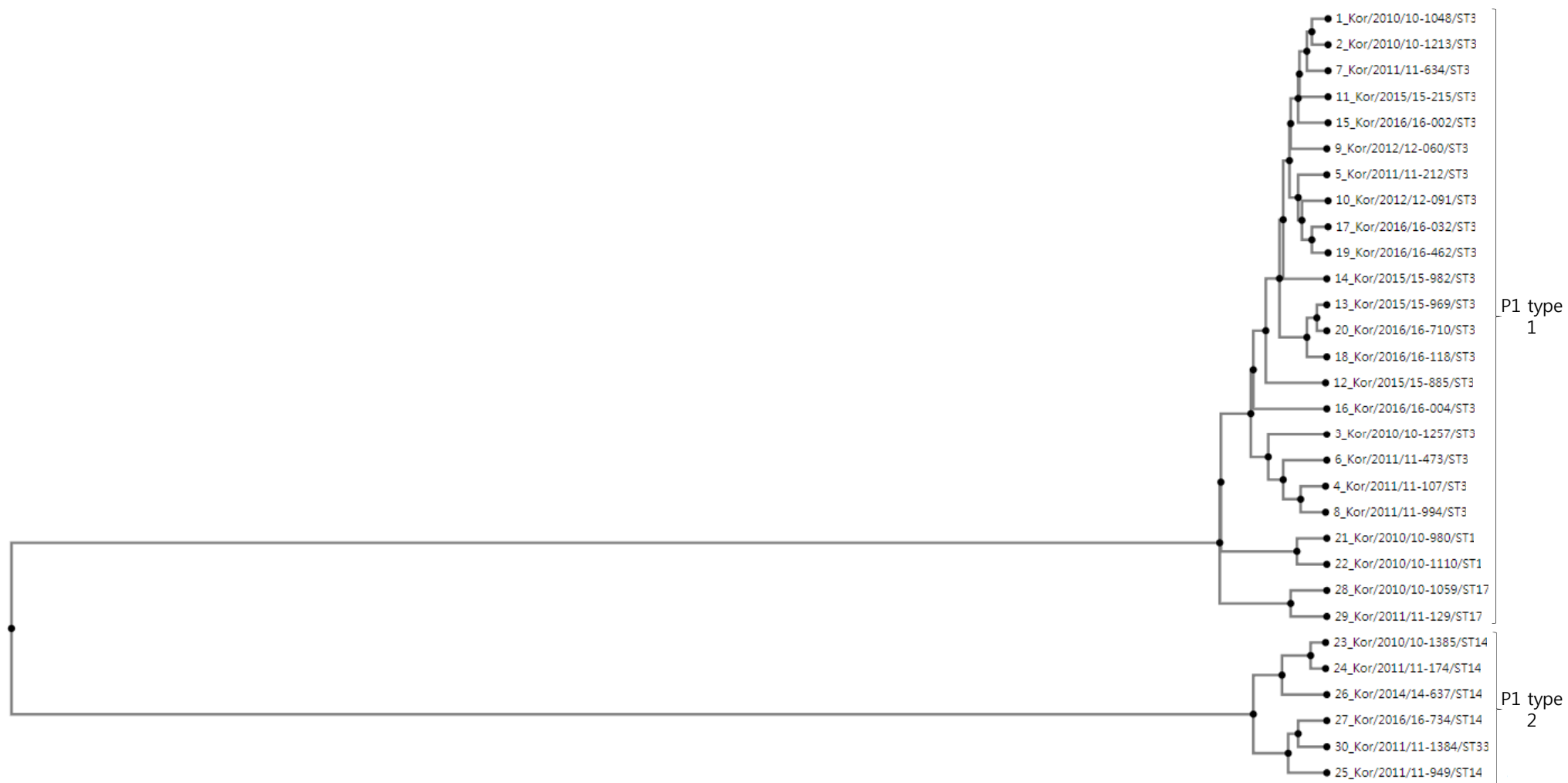

0.01

Supplement: Supplementary file 2 — Additional file 2: Phylogenetic tree based on whole genome alignment of the 30 sequenced strains. [file 12864_2019_6306_MOESM2_ESM.pdf]
